# Characterization of the Ca^2+^-coordination structures of L- and T-plastins in combination with their synthetic peptide analogs by FTIR spectroscopy

**DOI:** 10.1038/s41598-019-40889-9

**Published:** 2019-03-12

**Authors:** Masayuki Nara, Hisayuki Morii, Takashi Shimizu, Hiroto Shinomiya, Yuka Furuta, Kenichi Miyazono, Takuya Miyakawa, Masaru Tanokura

**Affiliations:** 1 Department of Chemistry, College of Liberal Arts and Sciences, Tokyo Medical and Dental University, Chiba, 272-0827 Japan; 2 National Institute of Advanced Industrial Science and Technology (AIST), Ibaraki, 305-8566 Japan; 3 Department of Medicine, Ehime University, Ehime, 791-0295 Japan; 4 Present Address: Ehime Prefectural Institute of Public Health and Environmental Science, Ehime, 790-0003 Japan; 5 Department of Applied Biological Chemistry, Graduate School of Agricultural and Life Sciences, University of Tokyo, Tokyo, 113-8657 Japan

## Abstract

FTIR spectroscopy was employed to characterize the coordination structures of divalent cations (M^2+^ = Ca^2+^ or Mg^2+^) bound by L- and T-plastins, which contain two EF-hand motifs. We focused on the N-terminal headpieces in the L- and T-plastins to analyze the regions of COO^−^ stretching and amide-I in solution. The spectral profiles indicated that these headpieces have EF-hand calcium-binding sites because bands at 1551 cm^−1^ and 1555 cm^−1^ were observed for the bidentate coordination mode of Glu at the 12th position of the Ca^2+^-binding site of Ca^2+^-loaded L-plastin and T-plastin, respectively. The amide-I profile of the Mg^2+^-loaded L-plastin headpiece was identical with that of the apo L-plastin headpiece, meaning that L-plastin has a lower affinity for Mg^2+^. The amide-I profiles for apo, Mg^2+^-loaded and Ca^2+^-loaded T-plastin suggested that aggregation was generated in protein solution at a concentration of 1 mM. The implications of the FTIR spectral data for these plastin headpieces are discussed on the basis of data obtained for synthetic peptide analogs corresponding to the Ca^2+^-binding site.

## Introduction

**Figure 1:**
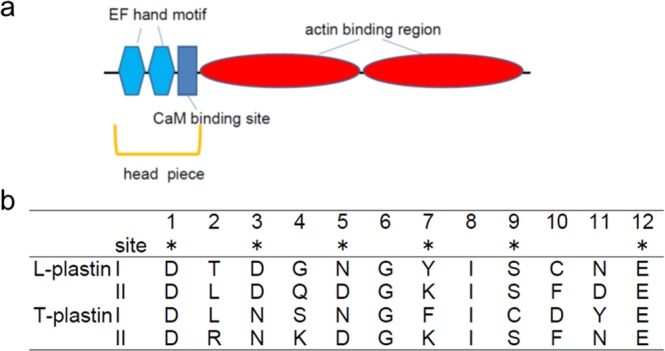
(**a**) Schematic structures of L- and T-plastins and (**b**) amino acid sequences for the two Ca^2+^-binding sites in the L- and T-plastin headpieces.

Plastins are known to be a family of actin-binding proteins that are evolutionarily conserved from yeast to mammalian cells^1^. These proteins contain two Ca^2+^ binding sites, one calmodulin (CaM) binding domain, and two actin-binding domains (Fig. 1a). The two calmodulin-like Ca^2+^-binding domains in plastins, so-called EF-hand motifs^2,3^, suggested that Ca^2+^ could regulate actin-binding or other functions of plastins^1^. In human, three isoforms have been characterized: L-plastin, T-plastin and I-plasin^1^. Human L-plastin is the only isoform that possesses all the conserved amino acids essential for Ca^2+^-binding and bundles actin filaments in a strictly Ca^2+^-regulated manner. The bundling activity of I-plastin is inhibited by Ca^2+^ (ref.^4^). The sensitivity of T-plastin for Ca^2+^ is lower than that of L-plastin. In mice, two isoforms, L-plastin and T-plastin, have so far been identified^5–8^. L-plastin isoform is expressed in leukocytes of normal cells and in many types of cancer cells, whereas the T-plastin isoform is constitutively expressed in epithelial and mesenchymal cells of solid tissues. The two isoforms differ in 21% of amino acid sequences. The actin-bundling activity of plastins was demonstrated to be regulated by Ca^2+^ through N-terminal EF-hand Ca^2+^-binding domains. In our previous study, it was indicated that mice L- and T-plastin headpieces changed their structures in response to Ca^2+^, but that the sensitivity to Ca^2+^ was higher in the L-plastin headpiece compared to the T-plastin headpiece in analyses carried out using various spectroscopic methods, gel-filtration chromatography, and isothermal titration calorimetry^9^. These results suggest that L-plastin is suitable for dynamic rearrangement of cytoskeletons, while T-plastin is suitable for maintaining static cytoskeletons^9^.

In the present study, Fourier transform infrared (FTIR) spectroscopy was employed to study the coordination structures of the divalent cation (M^2+^ = Mg^2+^ or Ca^2+^) bound in mice L- and T-plastin headpieces, each of which contains two EF-hand Ca^2+^-binding sites (Fig. 1b)^9^. The regions of COO^−^ antisymmetric and symmetric stretches provide information regarding the modes of coordination of a COO^−^ group to a metal ion^10–16^. The results showed that these headpieces have Ca^2+^ affinity in common with EF-hand calcium binding sites and a lower affinity for Mg^2+^. The amide-I profiles suggested that the T-plastin headpiece was more aggregable than the L-plastin headpiece. The implications of the FTIR spectral data for these plastin headpieces are discussed on the basis of the data obtained for the synthetic peptide analogs corresponding to a Ca^2+^-binding site.

## Results and Discussion

### FTIR spectra of L-plastin headpiece

**Figure 2:**
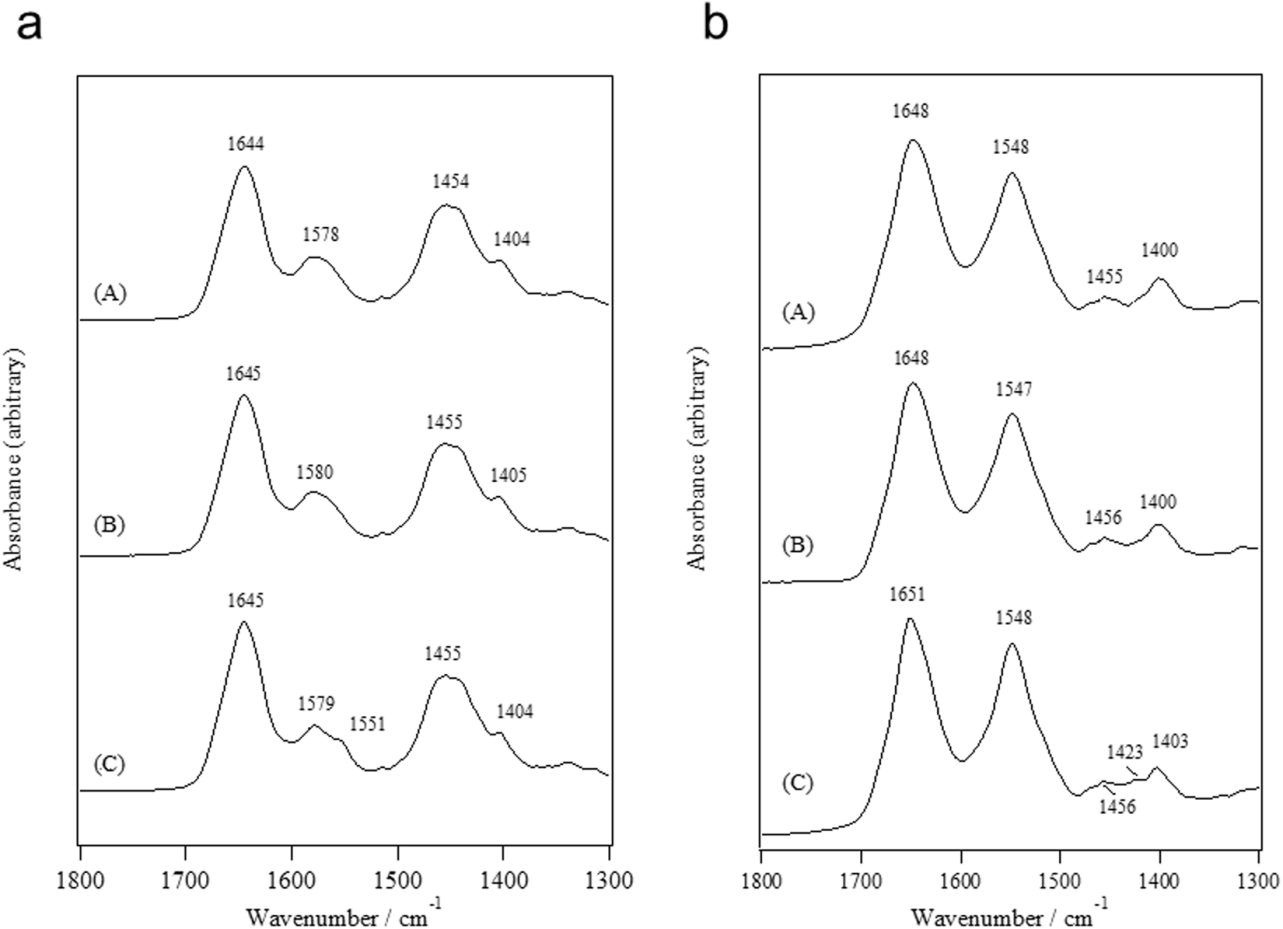
ATR-FTIR spectra for the L-plastin headpiece in the (A) apo, (B) Mg^2+^-loaded and (C) Ca^2+^-loaded states (**a**) in the D_2_O solutions and (**b**) in the H_2_O solutions.

Figure 2a shows the FTIR spectra for an L-plastin headpiece in the apo (M^2+^-free) and Mg^2+^ and Ca^2+^-loaded states in D_2_O solution in the wavenumber range of 1800–1300 cm^−1^. We observed the bands due to amide-I’, COO^−^ antisymmetric stretch, amide-II’, and COO^−^ symmetric stretch from the higher wavenumber side in Fig. 2a(A–C). A slight difference was detected in the region of COO^−^ antisymmetric stretching: a shoulder at approximately 1551 cm^−1^ appeared in the Ca^2+^-loaded state, although such a shoulder was not observed in the apo and Mg^2+^-loaded states.

Figure 2b also shows the FTIR spectra for the samples in H_2_O solution, where the contribution of the buffer has already been eliminated by subtracting the spectrum of the buffer, as mentioned in the Experimental section. The spectra indicated bands due to amide-I, amide-II, CH_2_ bending, and COO^−^ symmetric stretching from the higher wavenumber side. For the spectra obtained in H_2_O solution, the COO^−^ antisymmetric stretching mode is overlapped with the amide II mode, and therefore, we cannot extract the information about the COO^−^ antisymmetric stretch. However, the band at 1423 cm^−1^ was clearly observed only in the Ca^2+^-loaded state, which was thought to reflect the interaction of Ca^2+^ with the side-chain COO^−^ groups. The second-derivative spectra provide information in more detail regarding the spectral differences.

**Figure 3:**
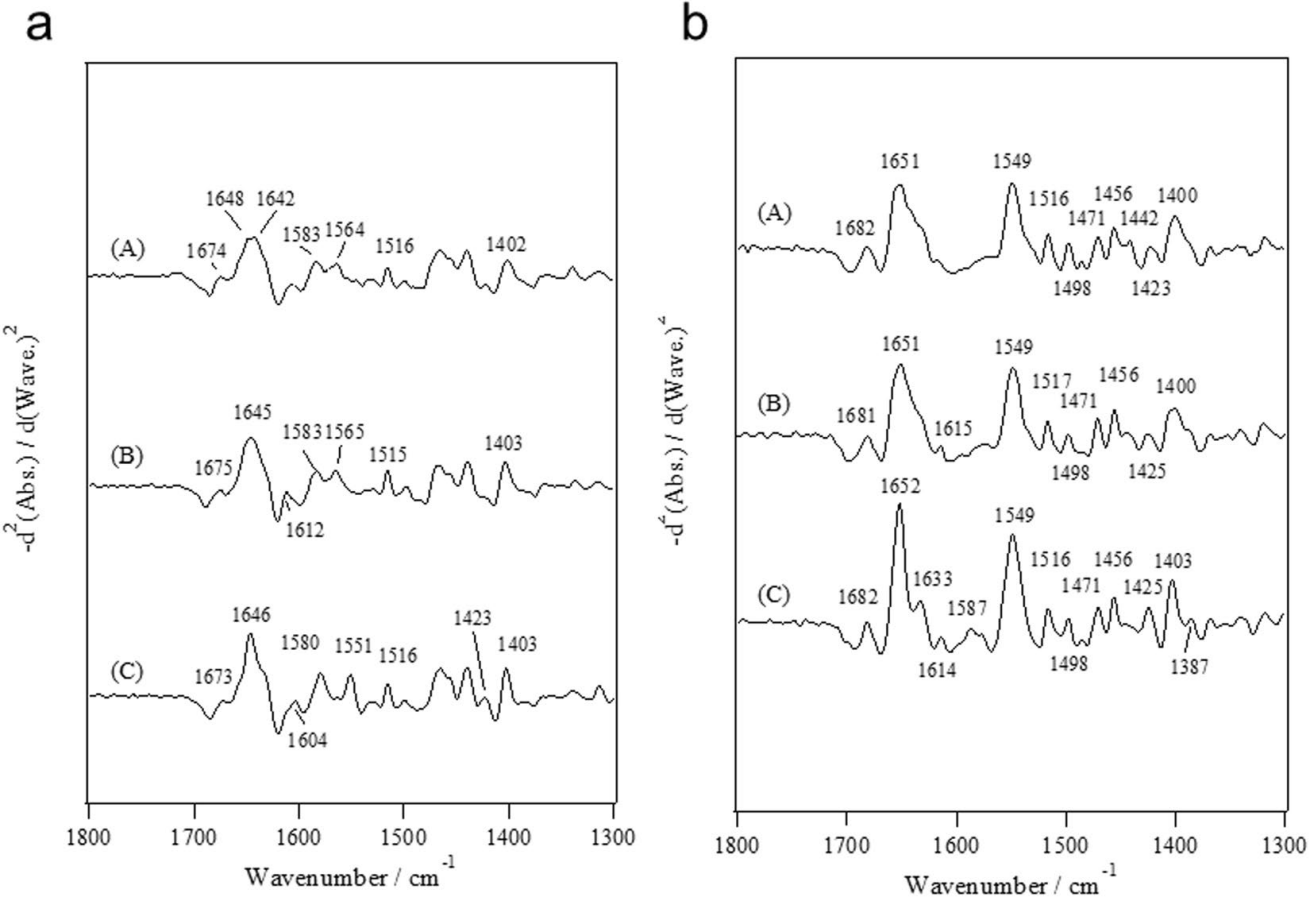
Second-derivative spectra for the L-plastin headpiece in the (A) apo, (B) Mg^2+^-loaded and (C) Ca^2+^-loaded states (**a**) in the D_2_O solutions and (**b**) in the H_2_O solutions.

In Fig. 3, the second-derivative spectra corresponding to the data shown in Fig. 2 are shown. From the region of the COO^−^ antisymmetric stretch, information regarding the coordination modes of the COO^−^ groups to the metal ions such as bidentate or pseudo-bridging modes is obtained^10–17^. The bands at 1583 cm^−1^ and 1564 cm^−1^ in the apo as well as Mg^2+^-loaded states (Fig. 3a(A,B)) were very close to the band at 1585 cm^−1^ due to β-COO^−^ of Asp and the band at 1565 cm^−1^ due to γ-COO^−^ of Glu, respectively^14–16^. The band at 1612 cm^−1^ was slightly stronger in the Mg^2+^-loaded state in comparison with that in the apo state. We observed the bands at 1604, 1580, and 1551 cm^−1^ in the Ca^2+^-loaded state where the band at 1551 cm^−1^ was undoubtedly due to the sidechain COO^−^ group binding to Ca^2+^ in the bidentate coordination mode^15,16^. In the COO^−^ symmetric stretch region, two bands at 1423 and 1403 cm^−1^ were detected, although one band at 1402 cm^−1^ was found only for the apo state.

The COO^−^ symmetric stretching region for the L-plastin headpiece in H_2_O solution also provides information regarding the coordination modes of the COO^−^ groups to the metal ions despite other vibrational modes such as the CH_2_ bending mode contributing to this region^15,16^. The amplitude at 1425 cm^−1^, which corresponds to the band at 1423 cm^−1^ in Fig. 2b(C), was stronger in the Ca^2+^-loaded state than in the apo and Mg^2+^-loaded states, which undoubtedly reflects the coordination modes of the COO^−^ groups to the metal ions as pseudo-bridging and/or bidentate modes. It is noted that the band at 1587 cm^−1^ for the L-plastin headpiece in the Ca^2+^-loaded state in H_2_O solution may reflect the coordination modes of COO^−^ groups to Ca^2+^ in the pseudo-bridging mode because the corresponding band in D_2_O solution was stronger in the Ca^2+^-loaded state than in the apo state^18^.

We also refer to the amide-I region because the spectral profiles for the Ca^2+^-loaded state in D_2_O and H_2_O solution were, respectively, different from those obtained for the apo and Mg^2+^-loaded states. The main peak position for amide-I and amide-I’ was the same among the apo, Mg^2+^-loaded and Ca^2+^-loaded states, but the bandwidth at the 1652 cm^−1^ band for the Ca^2+^-loaded state was clearly narrower than for the apo and Mg^2+^-loaded states (Fig. 3b). This spectral difference may reflect a conformational difference such as α-helix formation induced by Ca^2+^ binding. The bands at approximately 1682 cm^−1^ and 1633 cm^−1^ are assigned to a β-sheet conformation according to the empirical assignment of proteins^19,20^. The band at 1633 cm^−1^ was thought to not be induced by Ca^2+^ binding because a shoulder at 1633 cm^−1^ was also observed in the apo and Mg^2+^-loaded states and because the amplitude at approximately 1682 cm^−1^ was observed to be constant for these states.

### FTIR spectra for the T-plastin headpiece

**Figure 4:**
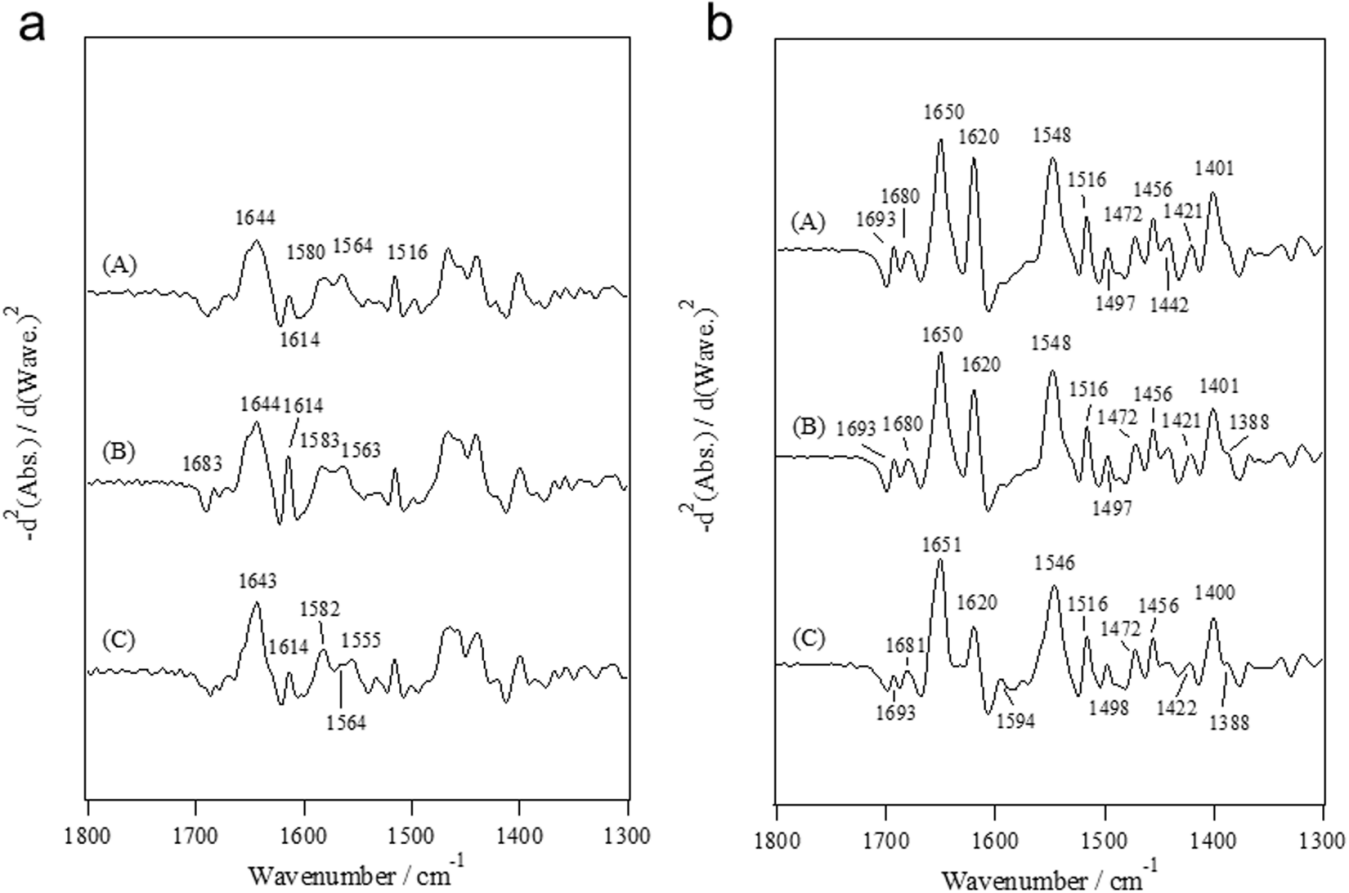
Second-derivative spectra for the T-plastin headpiece in the (A) apo, (B) Mg^2+^-loaded and (C) Ca^2+^-loaded states (**a**) in the D_2_O solutions and (**b**) in the H_2_O solutions.

Figure 4 depicts the second-derivative spectra for a T-plastin headpiece in the apo, Mg^2+^-loaded and Ca^2+^-loaded states in D_2_O and H_2_O solutions. Here, we left out the Attenuated total reflection (ATR) spectra for the T-plastin headpiece since the second-derivative spectra provide information regarding the coordination structure, as well as the conformational changes induced by M^2+^ in detail, as described in the FTIR section for the L-plastin headpiece. In the COO^−^ antisymmetric region, two bands at 1580 and 1564 cm^−1^ in the apo state, two bands at 1583 and 1563 cm^−1^ in the Mg^2+^-loaded state and three bands at 1582, 1564 and 1555 cm^−1^ in the Ca^2+^-loaded state were observed (Fig. 4a). The band at 1580 cm^−1^ in the apo state (Fig. 4a(A)) showed a 5 cm^−1^ downshift from the ionic Asp (1585 cm^−1^)^14–16^. The band at 1555 cm^−1^ in the Ca^2+^-loaded state (Fig. 4a(C)) was due to the side-chain COO^−^ groups binding to Ca^2+^ in the bidentate coordination mode^15,16^ but was 4-cm^−1^ higher than the corresponding band (1551 cm^−1^) for the L-plastin headpiece. In the region of the COO^−^ symmetric stretch, the bands at 1421 and 1400 cm^−1^ in D_2_O solution and the bands at approximately 1422 and 1401 cm^−1^ in H_2_O solution were observed.

The band at 1614 cm^−1^ was due to amide-I’ rather than the COO^−^ antisymmetric stretch (Fig. 4a) because the corresponding bands were observed at 1620 cm^−1^ in H_2_O solution (Fig. 4b), which moved together with the band at 1693 cm^−1^. The bands at 1693 and 1620 cm^−1^ were probably due to intermolecular interactions such as intermolecular β-strand since this protein seemed to easily aggregate with a spectral profile similar to that observed for a denatured protein^19,20^.

### CD spectra for plastin headpieces

**Figure 5:**
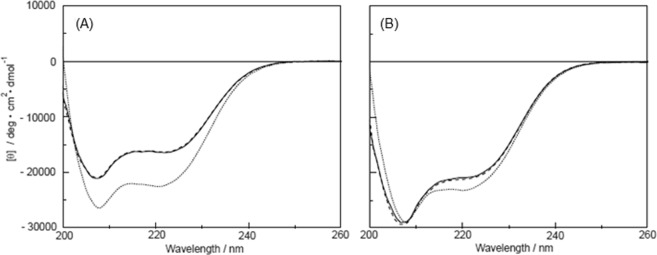
CD spectra for the headpiece from (**A**) L-plastin or (**B**) T-plastin in the apo (−), Mg^2+^(−), and Ca^2+^(······) states.

The effects of Mg^2+^- and Ca^2+^-binding in the headpieces were analyzed also by CD spectroscopy. The CD spectrum in each state showed two troughs around 208 and 222 nm (Fig. 5). For the L-plastin headpiece (Fig. 5(A)), both peaks at 208 nm and 222 nm in the CD spectra were shifted toward more negative values due to Ca^2+^-binding from apo and Mg^2+^-loaded states. On the other hand, the T-plastin headpiece showed a spectral change with an increasingly negative value only around the peak at 222 nm (Fig. 5(B)). These results suggest that the two headpieces are folded as a single polypeptide and are rich in α-helices. No change occurred due to the presence of Mg^2+^ on either of the headpieces, whereas some changes occurred due to Ca^2+^ -binding on both the peptides^9^. The secondary structural change induced by Ca^2+^-binding was greater in the L-plastin headpiece than in the T-plastin headpiece. Therefore, the increase in the α-helix content in the L-plastin headpiece due to Ca^2+^-binding should be larger than that in the T-plastin headpiece. This result was consistent with that data obtained using FTIR spectroscopy because these headpieces had a Ca^2+^ affinity in common with EF-hand calcium binding sites and less affinity for Mg^2+^; therefore, Mg^2+^ does not induce a conformational change in the headpieces.

### FTIR spectra for the synthetic peptide analogs of the Ca^2+^-binding sites

**Figure 6:**
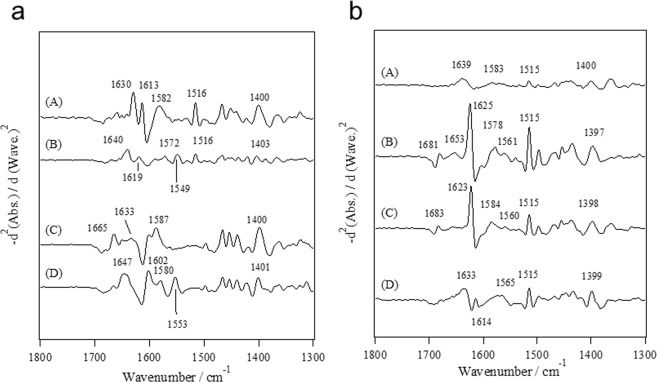
Second-derivative spectra for synthetic peptide analogs of (A,B) the Ca^2+^-binding site I and (C,D) the Ca^2+^-binding II of (**a**) L- plastin and (**b**) T-plastin. (A) and (C) are in the apo state and (B) and (D) are in the Ca^2+^-loaded state.

Figure 6 depicts the second-derivative spectra for 17-residue synthetic peptide analogs of the Ca^2+^-binding sites I and II of the L- and T-plastins in the wet film under D_2_O vapor atmosphere because the absorbance of the peptide in solution was too weak to analyze the amide I’ and COO^−^ stretching bands in detail. The bands at 1549 and 1553 cm^−1^ were detected for the peptide analogs corresponding to the site I and II of L-plastin in the Ca^2+^-loaded state, respectively (Fig. 6a(B,D)). Therefore, we confirmed that the COO^−^ of Glu at the 12th position is bound to Ca^2+^ in the bidentate coordination mode^15,16^. Meanwhile, the spectral profiles for the peptide analogs corresponding to the site I and II of T-plastin were quite different from those obtained for the T-plastin headpiece. Bands at 1561 and 1565 cm^−1^ were detected for the peptide analogs corresponding to sites I and II of T-plastin, respectively (Fig. 6b(B,D)), while a band at 1555 cm^−1^ was observed for the T-plastin headpiece in the Ca^2+^-loaded state (Fig. 4a(C)). The bands at 1681 and 1625 cm^−1^ in the amide I’ region (Fig. 6b(B)) suggested that the peptide analogs aggregated in the Ca^2+^-loaded state and that this aggregation disturbed the affinity of them for Ca^2+^. The same profile was also observed for the peptide analog corresponding to site II of T-plastin in the apo state (Fig. 6b(C)). Bands at 1681 and 1625 cm^−1^ were not observed for the Ca^2+^-loaded state in Fig. 6b(D), which suggested that Ca^2+^ reduced the aggregation of the peptide. We attempted to reduce the aggregation of site II of T-plastin by substitution of amino acid residue. At the present stage, we were not able to obtain information regarding the Ca^2+^-coordination structure for site II of T-plastin. However, as for site I of plastin T, we found that the mutant 17-residue peptide (C9K), where the cysteine was substituted for lysine at the 9^th^ position, did not aggregate. The FTIR spectra for the C9K peptide showed a band at 1560 cm^−1^ in the Ca^2+^-loaded state (data not shown), suggesting that the COO^−^ group of Glu at the 12^th^ position is bound to Ca^2+^ in the mode of pseudo-bridging coordination rather than in the mode of bidentate coordination.

## Conclusions

The results obtained using the synthetic peptide analogs suggested that the lower sensitivity to Ca^2+^ in the T-plastin headpiece may be related to the susceptibility to aggregation for the two Ca^2+^-binding sites. ATR-FTIR spectroscopy in combination with the use of a synthetic peptide analog approach is promising for understanding the correlation of Ca^2+^-binding coordination and the aggregation of Ca^2+^ binding proteins.

## Materials and Methods

### Sample preparation for plastin headpieces

Hexahistidine (His_6_)-tagged L- and T-plastin headpieces (L-plastin Δ1‒100 and T-plastin Δ1‒103) were expressed using a modified pET28a vector harboring their DNA sequences and *Escherichia coli* BL21(DE3) according to a previous report^9^. The expressed proteins were purified using Ni-NTA agarose (Qiagen). After cleaving the His_6_-tag on the resin with AcTEV protease, the eluted plastin headpieces were treated with trichloroacetic acid to remove contaminating Ca^2+^ ions^21^. Further purification was performed by anion-exchange chromatography and size-exclusion chromatography with Resource-Q and Superdex 75 columns (GE Healthcare), respectively.

### Sample preparation for the synthetic peptide analogs

We synthesized 17-residue peptide analogs corresponding to the two Ca^2+^-binding sites in the L- and T-plastins, as listed in Table 1, because the 17-residue peptide analogs for loop-helix F are the minimum number required for the Ca^2+^-binding property for site III of troponin C and site IV of akazara scallop troponin C^22,23^. The peptides were synthesized by the solid-phase method based on the Fmoc strategy^17,22,23^. A Fmoc-NH-SAL-PEG resin (Watanabe Chem.) solid-phase support containing the 4-[(2’,4’-dimethoxyphenyl) *N*-Fmoc-aminomethyl]phenoxyacetamido group, named Rink-amide linker^24^, was used to provide the peptides with *C*-terminal amide. The peptide chain was constructed in a stepwise manner for respective amino acid residues. The coupling reaction of a side-chain protected Fmoc-amino acid was carried out with an equimolar reagent system of HBTU-HOBt in DMF containing a double equivalence of *N*-methyl morpholine. In every synthetic cycle, Fmoc-protecting groups of the elongating peptide chains were deblocked with mixed reagents of piperidine-DBU-HOBt, with concentrations of 8%(v/v), 2%(v/v), and 2.5%(w/v) in DMF, respectively. The additive HOBt was used to suppress the side reaction of the aspartic residue caused by piperidine^25^. After completion of elongation, the peptides were harvested by cleavage reaction with trifluoroacetic acid (TFA) containing EDT (4%), TIPS (6%), and water (2%). The crude peptides were dissolved in a LiCl (4%) solution of DMF and purified by reverse-phase HPLC. The molecular weights of the peptides were confirmed by MALDI-TOFMS with AXIMA (Shimadzu). The TFA carried-over from HPLC purification was completely removed with a size-exclusion column, PD-10 (GE Healthcare), in 0.1 M ammonium bicarbonate buffer solution (pH 8.5) containing 0.1 M KCl, because TFA causes disruptive overlapping of the FTIR signals^22,23^. Finally, the collected fractions of peptides were desalted with PD-10 in pure water.

**Table 1:** The amino acid residue for the Ca^2+^-binding site for T- and L-plastins.

	site		*		*		*		*		*			*					
T-plastin	**I**	Ac-	D	L	N	S	N	G	F	I	C	D	Y	E	L	H	E	L	F
	**II**	Ac-	D	R	N	K	D	G	K	I	S	F	N	E	F	V	Y	I	F
L-plastin	**I**	Ac-	D	T	D	G	N	G	Y	I	S	C	N	E	L	N	D	L	F
	**II**	Ac-	D	L	D	Q	D	G	K	I	S	F	D	E	F	I	K	V	F

C: cysteine as Cys(CH_3_).

### FTIR measurements

Most of the experiments described for FTIR measuremrnts were performed in the same manner as in our previous works^17,26,27^. ATR-FTIR measurements were carried out for the L- and T- plastin headpieces at room temperature using a Perkin-Elmer Spectrum-One Fourier transform infrared spectrometer equipped with an ATR unit and an MCT detector with a resolution of 2 cm^−1^ ^27^. Interferograms from 200 scans were averaged for the series of measurements for L-plastin. On the other hand, interferograms from 500 scans were averaged for the series of measurements for the T-plastin headpiece since the sample concentration was lower (approximately half of the L-plastin headpiece concentration) due to partial aggregation^17^. Dry air gas was constantly pumped into the ATR unit of the spectrometer to suppress water vapor^17^. Approximately 10 μl of a sample solution was placed onto a Diamond/ZnSe 1-reflection top–plate (Perkin-Elmer). ATR-FTIR spectra for the solvents (buffer solutions) were measured in the same way. The treatment and analyses of the ATR-FTIR spectra have been described previously^17,27^. For the synthetic peptide analogs for the calcium binding sites of L- and T-plastins, ATR-FTIR measurements were also carried out for samples in a wet film under D_2_O vapor atmosphere to determine the absorbance intensity of the amide I’ and COO^−^ stretching modes^17^.

### CD measurements

CD spectra were measured using a spectrometer J-720 (Jasco) at room temperature. The acquisition parameters were as follows: resolution, 0.2 nm; speed, 50 nm/min; response time, 2 s; bandwidth, 1 nm; and scan, 10. The 0.02 mM protein solution was prepared in 10 mM MOPS-KOH (pH 6.8), 100 mM KCl, and 0.05 mM EDTA for the M^2+^-free state and the same composition containing 2 mM MCl_2_, and for the M^2+^-loaded state. Each spectrum was subtracted with that from the buffer.

## References

[1] Deranote V, Vandekerckhove J, Gettemans J (2005). Plastins: versatile modulators of actin organization in (patho) physiological cellular processes. Acta Pharm. Sinica.

[2] Kretsinger RH, Nockolds CE (1973). Carp muscle calcium-binding protein. 2. Structure determination and general description. J. Biol. Chem..

[3] Moews PC, Kretsinger RH (1975). Refinement structure of carp muscle calcium-binding parvalbumin by model-building and difference Fourier-analysis. J. Mol. Biol..

[4] Lin CS, Shen WY, Chen ZP, Tu YH, Matsudaira P (1994). Identification of I-plastin, a human fimbrin isoform expressed in intestine and kidney. Mol. Cell. Biol..

[5] Shinomiya H (2003). Preparation and characterization of recombinant murine p65/L-plastin expressed in *E*. *coli* and high-titer antibodies against the protein. Biosci. Biotechnol. Biochem..

[6] Toyooka K (2006). Generation and characterization of monoclonal antibodies that specifically recognize p65/L-plastin isoform but not T-plastin isoform. Biosci. Biotechnol. Biochem..

[7] Shinomiya H, Shinjo M, Fengzhi L, Asano Y, Kihara H (2007). Conformational analysis of the leukocyte-specific EF-hand protein p65/ L-plastin by X-ray scattering in solution. Biophys. Chem..

[8] Hagiwara M (2011). Interaction of activated Rab5 with actin-bundling proteins, L- and T-plastin and its relevance to eudocytic functions in mammalian cells. Biochem. Biophys. Res. Comm..

[9] Miyakawa T (2012). Different Ca^2+^-sensitivities between the EF-hands of T- and L-plastins. Biochim. Biophys. Res. Comm..

[10] Deacon GB, Phillips RJ (1980). Relationships between the carbon-oxygen stretching frequencies of carboxylato complexes and the type of carboxylate coordination. Coord. Chem. Rev..

[11] Nakamoto, K. Infrared and Raman spectra of inorganic and coordination compounds, Part B, 5th edn., 57–62 (Wiley, 1997).

[12] Tackett JE (1989). FT-IR characterization of metal acetates in aqueous-solution. Appl. Spectrosc..

[13] Nara M, Torii H, Tasumi M (1996). Correlation between the vibrational frequencies of the carboxylate group and the types of its coordination to a metal ion: an ab initio molecular orbital study. J. Phys. Chem..

[14] Nara M (1994). Infrared studies of interaction between metal ions and Ca^2+^-binding proteins. Marker bands for identifying the types of coordination of the side-chain COO^−^ groups to metal ions in pike parvalbumin (pI = 4.10). FEBS Lett..

[15] Nara M, Tanokura M (2008). Infrared spectroscopic study of the metal-coordinations structures of calcium-binding proteins. Biochem. Biophys. Res. Comm..

[16] Nara M, Morii H, Tanokura M (2013). Coordination to divalent cations by calcium-binding proteins studied by FTIR spectroscopy. Biochim. Biophys. Acta.

[17] Nara, M., Morii, H. & Tanokura, M. Coordination to divalent cations by calcium-binding proteins: from basics to medical applications in *Methods in Molecular Biology* (ed. Heizmann, C. W.) 127–134 (Springer Nature, 2019). 10.1007/978-1-4939-9030-6_9 30710271

[18] Mizuguchi M, Nara M, Kawano K, Nitta K (1997). FTIR study of the Ca^2+^-binding to bovine α-lactalbumin. Relationships between the type of coordination and characteristics of the bands due to the Asp COO^−^ groups in the Ca^2+^-binding site. FEBS Lett..

[19] Arrondo JLR, Goni FM (1999). Structure and dynamics of membrane proteins as studied by infrared spectroscopy. Prog. Biophys. Mol. Biol..

[20] Barth A, Zscherp C (2002). What vibrations tell us about proteins. Q. Rev. Biophys..

[21] Tanokura M, Yamada K (1984). Heat capacity and entropy changes of calmodulin induced by calcium binding. J. Biochem..

[22] Nara M, Morii H, Yumoto F, Kagi H, Tanokura M (2006). Fourier transform infrared spectroscopic study on the Ca^2+^-bound coordination structures of synthetic peptide analogues of the calcium-binding site III of troponin C. Biopolymers.

[23] Nara M (2006). Infrared spectroscopic study on Ca^2+^ binding to Akazara scallop troponin C in comparison with peptide analogues of its Ca^2+^ binding site IV. Vib. Spectrosc..

[24] Rink H (1987). Solid-phase synthesis of protected peptide fragments using a trialkoxy-diphenyl-methylester resin. Tetra. Lett..

[25] Dölling R (1994). Piperidine-mediated side product formation for Asp(OBut)-containing peptides. J. Chem. Soc. Chem. Commun..

[26] Yumoto F (2001). Coordination structures of Ca^2+^ and Mg^2+^ in Akazara scallop troponin C in solution. FTIR spectroscopy of side-chain COO^−^ groups. Eur. J. Biochem..

[27] Nara M, Morii H, Tanokura M (2013). Infrared study of synthetic peptide analogues of the calcium-binding site III of troponin C: the role of helix F of an EF-hand motif. Biopolymers.

